# Tissue-specific oxidative stress and bleomycin hydrolase signatures are associated with organ-specific fibrotic responses in a mouse model of systemic sclerosis

**DOI:** 10.3389/fcell.2026.1888823

**Published:** 2026-08-18

**Authors:** Rishabh Johri, Marianela Brizio, Maximilien Lora, Sydney Joy, James Martin, Inés Colmegna

**Affiliations:** 1 Department of Microbiology and Immunology, McGill University, Montreal, QC, Canada; 2 The Research Institute of the McGill University Health Centre (RI-MUHC), Montreal, QC, Canada; 3 Division of Experimental Medicine, McGill University, Montreal, QC, Canada; 4 Division of Rheumatology, McGill University Health Centre, Montreal, QC, Canada

**Keywords:** bleomycin, bleomycin hydrolase, fibrosis, inflammation, oxidative stress, scleroderma, systemic sclerosis

## Abstract

Systemic sclerosis (SSc) is a multisystem autoimmune disease characterized by inflammation, vasculopathy, and progressive fibrosis, yet the mechanisms underlying specific organ involvement are incompletely understood. Using the bleomycin–osmotic minipump (BLM-MP) mouse model, which recapitulates key clinical and histopathological features of SSc, we investigated whether tissue-specific oxidative stress responses and BLM metabolism are associated with divergent inflammatory and fibrotic remodeling across organs. Male C57BL/6 mice received continuous systemic infusion of BLM or saline for 7 days. Lungs, skin, kidneys, and liver were analyzed 14 and 28 days later by histopathology, collagen quantification, immunohistochemistry, and RT-qPCR. Systemic BLM exposure induced distinct organ-specific inflammatory, fibrotic and molecular responses. Progressive fibrosis and inflammatory infiltration developed predominantly in the lungs and skin, whereas the kidneys exhibited only mild focal cortical fibrosis and the liver remained largely unaffected. Fibrosis-prone organs displayed impaired antioxidant responses, characterized by reduced expression of superoxide dismutase 1 (*Sod1*), glutathione peroxidase 3 (*Gpx3*), and catalase (*Cat*), together with decreased expression of bleomycin hydrolase (*Blmh*), the enzyme responsible for BLM inactivation. In contrast, fibrosis-resistant organs exhibited preserved or increased expression of antioxidant enzymes and *Blmh* following BLM exposure, consistent with activation of protective redox and drug-clearance pathways. Across organs, lower antioxidant and *Blmh* expression was associated with greater inflammatory infiltration and collagen deposition. These findings identify a tissue-specific molecular signature with divergent inflammatory and fibrotic responses following systemic BLM exposure.

## 1 Introduction

Fibrosis is a pathological tissue repair response driven by chronic inflammation and repetitive tissue injury, characterized by persistent myofibroblast activation and excessive extracellular matrix (ECM) deposition (Rieder et al., 2025). Although fibrosis can affect virtually any organ, the extent and pattern of tissue involvement vary markedly, suggesting the existence of organ-specific protective and pathogenic mechanisms. Systemic sclerosis (SSc) is a prototypical multi-organ autoimmune disease characterized by immune dysregulation, vasculopathy, and progressive fibrosis (Volkmann et al., 2023). While cutaneous fibrosis is the defining clinical feature of SSc, interstitial lung disease (SSc-ILD) represents the leading cause of mortality, and fibrosis-related dysfunction of the renal, cardiovascular, and gastrointestinal systems contributes substantially to disease burden (Denton and Khanna, 2017; Distler et al., 2026). Fibrogenesis in SSc is driven by the interplay between chronic inflammation, oxidative stress, and dysregulated repair (Distler et al., 2026). Despite advances in understanding fibrogenic pathways, effective antifibrotic therapies remain limited, and the mechanisms that determine tissue-specific susceptibility to fibrosis remain incompletely defined (Distler et al., 2025).

The bleomycin–osmotic minipump (BLM-MP) model recapitulates key clinical and histopathological features of SSc including subpleural interstitial lung fibrosis and dermal fibrosis with skin lipoatrophy (Lee et al., 2014; Liang et al., 2015; Morozan et al., 2023). Although pulmonary and cutaneous manifestations have been well characterized, the response of other organs to sustained BLM exposure remains less well defined. In particular, little is known about the molecular programs that distinguish fibrosis-prone from fibrosis-resistant tissues. Because BLM exerts its cytotoxic effects primarily through oxidative stress (Dorr, 1992; Allawzi et al., 2019), and because both endogenous antioxidant capacity (Kim et al., 2018) and bleomycin hydrolase (BLMH)-mediated drug metabolism vary across tissues (Schwartz et al., 1999), we hypothesized that systemic BLM exposure induces tissue-specific antioxidant and *Blmh* expression signatures that are associated with divergent inflammatory and fibrotic remodeling across organs. To test this hypothesis, we comprehensively characterized the liver, kidneys, skin, and lungs following systemic BLM exposure and examined whether differences in canonical antioxidant responses and *Blmh* expression were associated with organ-specific inflammatory infiltration and fibrosis.

## 2 Materials and methods

### 2.1 BLM-MP mouse model

Mice were housed in a conventional animal facility at the Research Institute of the McGill University Health Centre (RI-MUHC). Animals were treated in accordance with the guidelines of the Canadian Council of Animal Care (CCAC) and the study protocol (MUHC-5964) was approved by the Animal Care Committee of McGill University. Osmotic minipumps containing 90 U/kg clinical grade bleomycin (BLM) or saline (PBS) were subcutaneously implanted into ten-week-old male C57BL/6 mice purchased from Jackson Laboratories as previously described (Morozan et al., 2023). Minipump contents were delivered as a continuous infusion at a rate of 0.5 μ L per hour for 7 days and removed on D10. Mice were sacrificed 14 and 28 days post implantation (n = 12 per group at each timepoint).

### 2.2 Histological assessment of fibrosis

Following euthanasia, the liver, kidneys, skin, and lungs were removed, washed with PBS, formaldehyde-fixed, paraffin-embedded, and sectioned as previously described (Morozan et al., 2023; Brizio et al., 2024). To observe collagen deposition and ECM organization, slides were stained with Picrosirius Red (PSR) and were analyzed with ImageJ. The ImageScope Positive Pixel Count algorithm (version 9) was used to detect the percent of positively stained tissue in a selected target region. To assess tissue structure, composition, and cellularity in the lungs, liver, and skin, Hematoxylin & Eosin (H&E) was performed while kidneys were stained with Periodic Acid Schiff (PAS) to highlight the glomerular basement membrane (Lai and Lü, 2012).

### 2.3 Immunohistochemistry (IHC)

Liver, kidney, lung, and skin sections (collected from the lower back, distal to the minipump implantation site) were cut at 4 µm, deparaffinized and rehydrated. Immunohistochemical staining was performed at the RI-MUHC Histology Platform using the Roche Discovery Ultra instrument. Following antigen retrieval for 32 min using ULTRA Cell Conditioning Solution (#5242452001), tissue sections were incubated with anti-CD45 antibody (Cell Signaling Technology, #70257) for 32 min at 37 °C. Sections were subsequently incubated with OmniMap anti-rabbit HRP secondary antibody (Roche, #760–4311) for 20 min at room temperature, and signal detection was performed using DAB chromogen (ChromoMap, Roche, #760–159). Slides were then counterstained with hematoxylin, dehydrated through graded alcohols, cleared, and cover slipped. Whole-slide digital images were acquired using an Aperio Turbo 2T scanner.

### 2.4 CD45 IHC analysis

CD45^+^ cells were quantified in scanned tissue sections using QuPath (v0.6.0) with both supervised classifier-based and threshold-based positive cell detection approaches (Bankhead et al., 2017). For the classifier-based method, a pixel classifier trained on annotated regions distinguished CD45^+^ cells from tissue artifacts using hematoxylin/DAB intensities and cell morphology. For the threshold-based method, nuclei were segmented by hematoxylin staining and classified as CD45^+^ based on DAB intensity. Watershed-based separation was applied to resolve adjacent cells. Consistent parameters were used within each organ, and CD45^+^ frequency was calculated as the percentage of CD45^+^ cells among total cells and averaged across multiple regions per slide. Analyses were independently validated by two observers.

### 2.5 Real time (RT)-qPCR

RNA was extracted from all tissues using the RNeasy Mini Kit (QIAGEN, #74104), except for skin samples, which were processed with the RNeasy Fibrous Tissue Mini Kit (QIAGEN, #74704). In all cases, 30 mg of tissue were used for extraction. cDNA was synthesized according to the manufacturer’s instructions with the QuantiTect Reverse Transcription Kit (QIAGEN, #205311), and RT-qPCR was performed using SYBR Green detection (QIAGEN, #208056) on a thermocycler. Relative gene expression was calculated using the ΔΔCT method. Primer sequences used for RT-qPCR are listed in Supplementary Table S1.

### 2.6 Statistical analysis

Normality was assessed using the Shapiro-Wilk test. Multiple group comparisons were performed using one-way ANOVA followed by Tukey’s *post hoc* test. For non-normally distributed datasets, non-parametric *post hoc* analyses were applied. A p value < 0.05 was considered statistically significant.

To correlate PSR and CD45 with *Col1a1*, *Sod1*, *Gpx3*, *Cat* and *Blmh* gene expression across organs, PSR and CD45 mean fold changes at D14 or D28 over baseline were calculated and Spearman rank correlations were computed across the eight organ/timepoint groups. Tests were adjusted using the Benjamini-Hochberg test. Statistical analyses and data visualization were performed using GraphPad Prism 11.0.1 (GraphPad Software, San Diego, CA, United States).

## 3 Results

### 3.1 Organ-specific fibrotic responses following systemic BLM exposure

**FIGURE 1:**
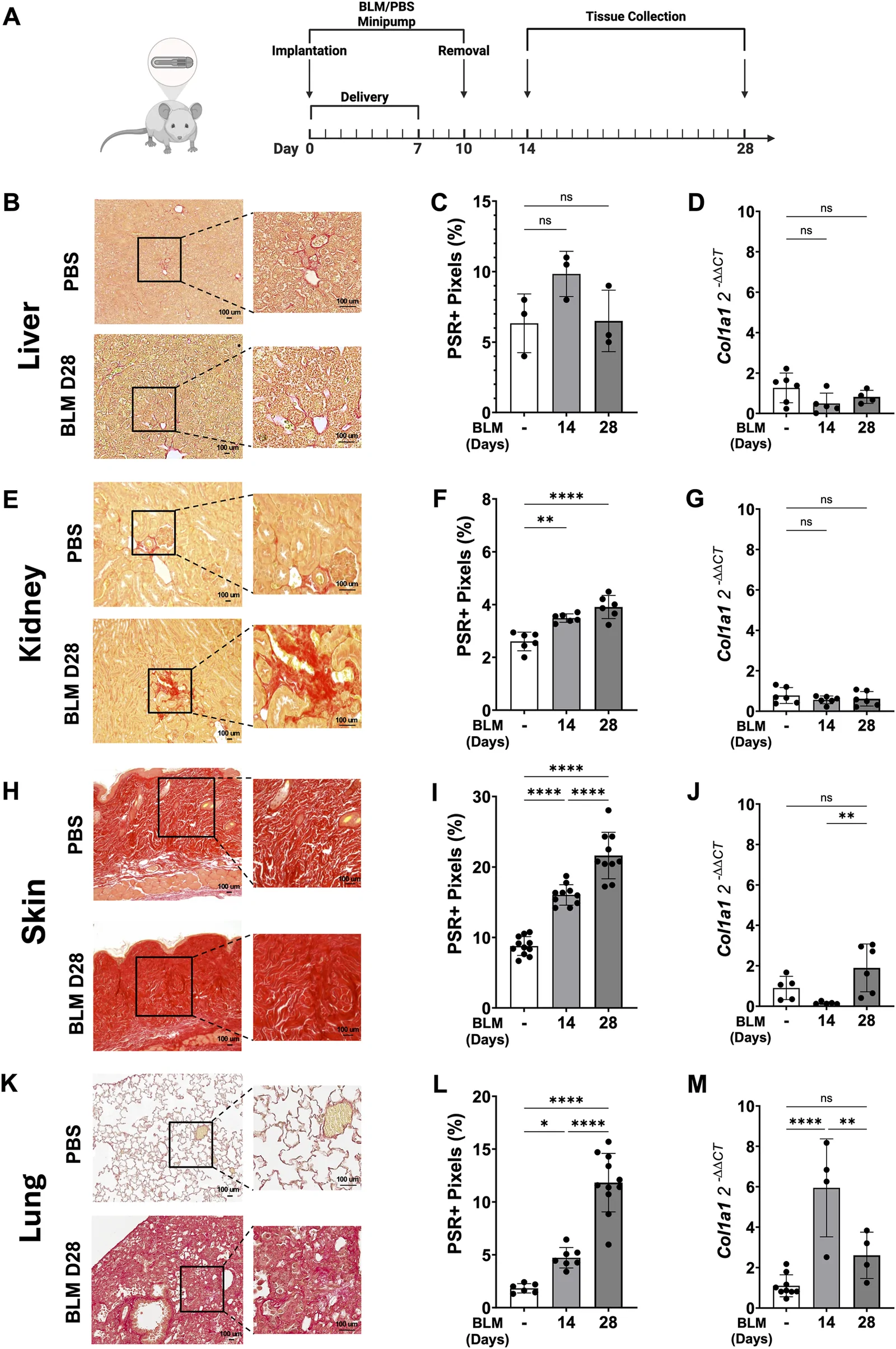
Tissue-specific fibrotic responses following systemic BLM exposure. **(A)** Schematic representation of the experimental design. Osmotic minipumps containing bleomycin (BLM) or phosphate-buffered saline (PBS) were implanted on D0 and delivered their contents continuously for 7 days before removal on D10. Tissues were collected on D14 and D28. Representative Picrosirius Red (PSR)-stained sections, quantification of collagen-positive area (% PSR-positive pixels), and Col1a1 mRNA expression in the **(B–D)** liver, **(E–G)** kidneys, **(H–J)** skin, and **(K–M)** lungs. Representative images were acquired at ×10 and ×40 magnification. Scale bar = 100 μm. Quantitative analyses were performed in PBS-MP controls and BLM-MP mice at D14 and D28. Relative Col1a1 expression was determined by RT-qPCR using the ΔΔCt method and normalized to PBS controls. Data are presented as mean ± SD. Statistical significance was determined by one-way ANOVA with Tukey’s *post hoc* test. ns, not significant; *p < 0.05; **p < 0.01; ***p < 0.001; ****p ≤ 0.0001.

To characterize organ-specific fibrotic responses following systemic BLM exposure, the liver, kidneys, skin and lungs were histologically evaluated 14 and 28 days after BLM-MP implantation (Figure 1A). Compared to PBS-MP controls, BLM-MP mice showed no histological evidence of liver fibrosis (PBS vs. BLM D14 vs. BLM D28, n=3 mean ± SD,: 6.33 ± 2.08 vs. 9.83 ± 1.61 vs. 6.50 ±2.18 ); (Figures 1B,C) and hepatic *Col1a1* expression remained unchanged (PBS vs. BLM D14 vs. BLM D28, n=4−6mean±SD : 1.26 ± 0.74 vs. 0.49 ± 0.51 vs. 0.82 ±0.33 ) (Figure 1D). In the kidneys, collagen deposition was mild and limited to focal interstitial tubular areas within the renal cortex (Figure 1E). Although Picrosirius Red-positive staining modestly increased following BLM exposure (PSR^+^ %, PBS vs. BLM D14 vs. BLM D28, n=6 mean ± SD: 2.60 ± 0.35 vs. 3.49 ± 0.16 vs. 3.91 ± 0.44) (Figure 1F), whole-kidney *Col1a1* transcript levels did not differ from controls (PBS vs. BLM D14 vs. BLM D28, n=6 mean ± SD: 0.78 ± 0.39 vs. 0.56 ± 0.08 vs. 0.62 ±0.36 ) (Figure 1G).

In contrast, progressive fibrotic remodeling developed in skin and lungs of BLM treated mice. Dermal collagen deposition increased at D14 and was further elevated at D28 (PSR^+^ %, PBS vs. BLM D14 vs. BLM D28, n=10−11 mean ± SD: 8.78 ± 1.33 vs. 16.03 ± 1.46 vs. 21.63 ± 3.29) (Figures 1H,I), whereas *Col1a1* transcript levels increased from D14 to D28 but remained comparable to controls (PBS vs. BLM D14 vs. BLM D28, n=5−6 mean ± SD: 0.90 ± 0.58 vs. 0.16 ± 0.07 vs. 1.82 ±1.30 ) (Figure 1J). Similarly, pulmonary fibrosis progressively increased over time, predominantly involving the subpleural interstitium by D28 (Figure 1K). Lung collagen deposition and *Col1a1* expression were increased at D14 and remained higher than controls at D28 (PSR^+^ %, PBS vs. BLM D14 vs. BLM D28, n=6−11 mean ± SD: 1.83 ± 0.44 vs. 4.72 ± 0.97 vs. 11.82 ± 2.77) (Figure 1L) (*Col1a1* PBS vs. BLM D14 vs. BLM D28, n=4−9 mean ± SD: 1.10 ± 0.54 vs. 5.94 ± 2.42 vs. 2.61 ± 1.14 (Figure 1M). Collectively, these findings demonstrate marked heterogeneity in tissue responses to systemic BLM exposure, with progressive fibrotic remodeling occurring predominantly in the skin and lungs, whereas the kidneys were minimally involved and the liver remained largely resistant.

### 3.2 Inflammatory cell infiltration accompanies early tissue remodeling following BLM exposure

**FIGURE 2:**
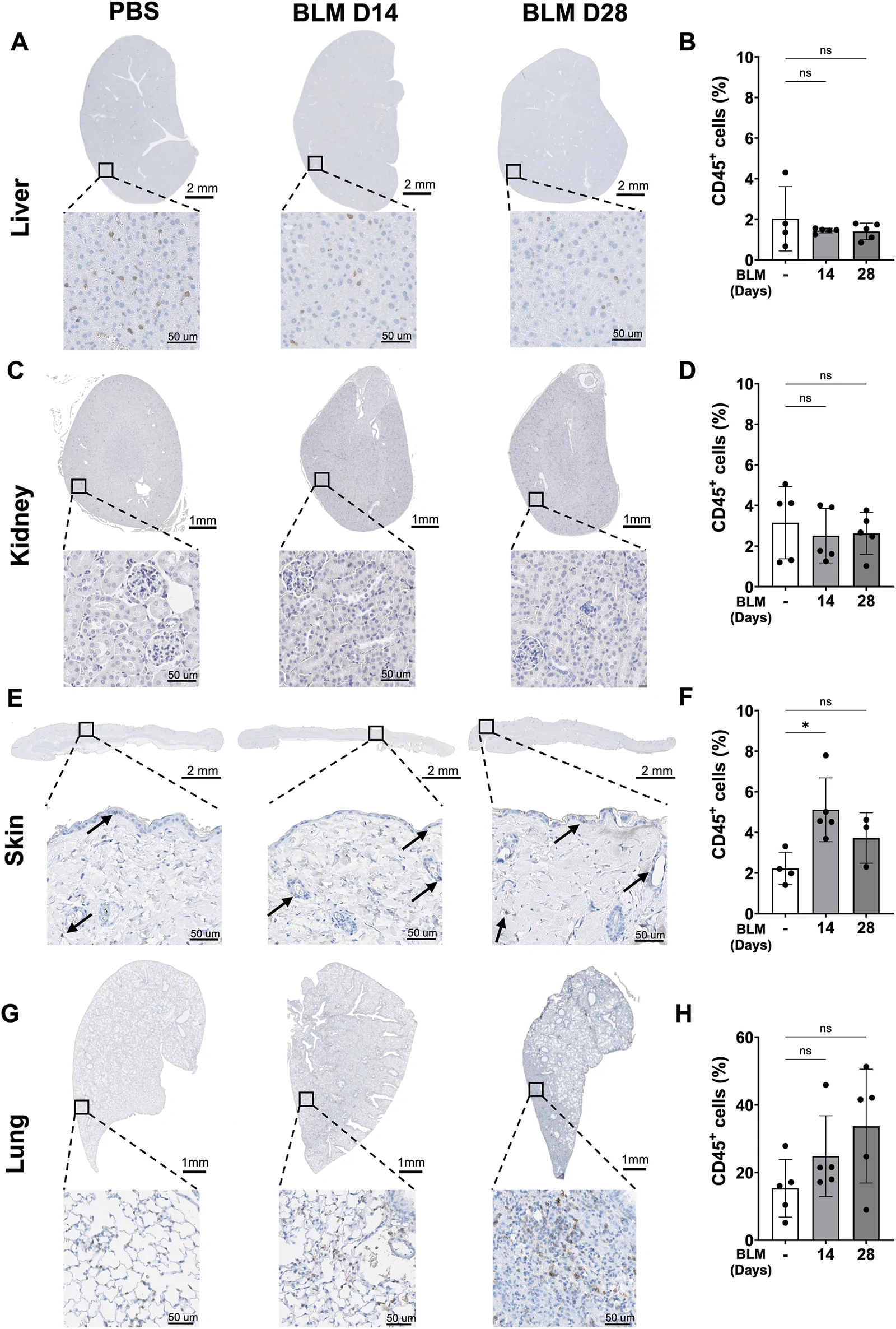
Tissue-specific inflammatory cell infiltration following systemic BLM exposure. **(A,C,E,G)** Representative CD45 immunohistochemical staining of the liver, kidneys, skin, and lungs from PBS-MP control and BLM-MP mice. **(B,D,F,H)** Quantification of CD45^+^ leukocyte frequency in the corresponding tissues. Whole-slide images and representative high-magnification regions (20×) are shown. CD45^+^ cell frequency is expressed as the percentage of total nucleated cells. Quantitative analyses were performed in PBS-MP controls and BLM-MP mice at D14 and D28. Data are presented as mean ± SD. Statistical significance was determined by one-way ANOVA with Tukey’s *post hoc* test. ns, not significant; *p < 0.05.

To assess inflammatory responses following systemic BLM exposure, CD45^+^ leukocyte infiltration was quantified in the liver, kidneys, skin, and lungs at D14 and D28. No differences in CD45^+^ cell frequency were observed in either the liver (CD45^+^ %, PBS vs. BLM D14 vs. BLM D28, n=4−5 mean ± SD: 2.03 ± 1.58 vs. 1.45 ± 0.12 vs. 1.40 ±0.41 ) (Figures 2A,B) or kidneys (CD45^+^ %, PBS vs. BLM D14 vs. BLM D28, n=5 mean ± SD: 3.15 ± 1.77 vs. 2.51 ± 1.34 vs. 2.63 ± 1.03(Figures 2C,D) relative to controls. In contrast, dermal CD45^+^ cell infiltration was increased at D14, but returned toward baseline by D28 in BLM-treated mice (CD45^+^ %, PBS vs. BLM D14 vs. BLM D28, n=3−5 mean ± SD: 2.23 ± 0.80 vs. 5.11 ± 1.57 vs. 3.73 ± 1.25 (Figures 2E,F). These findings indicate that inflammatory cell recruitment occurs early during dermal remodeling in this model. In the lungs, CD45^+^ cell frequencies were numerically higher following BLM exposure, however, these differences did not reach statistical significance and were associated with substantial inter-animal variability (CD45^+^ %, PBS vs. BLM D14 vs. BLM D28, n=5 mean ± SD: 15.3 ± 8.50 vs. 24.8 ± 12.0 vs. 33.7 ± 16.8 (Figures 2G,H).

### 3.3 Tissue-specific antioxidant responses are associated with divergent fibrotic remodeling

**FIGURE 3:**
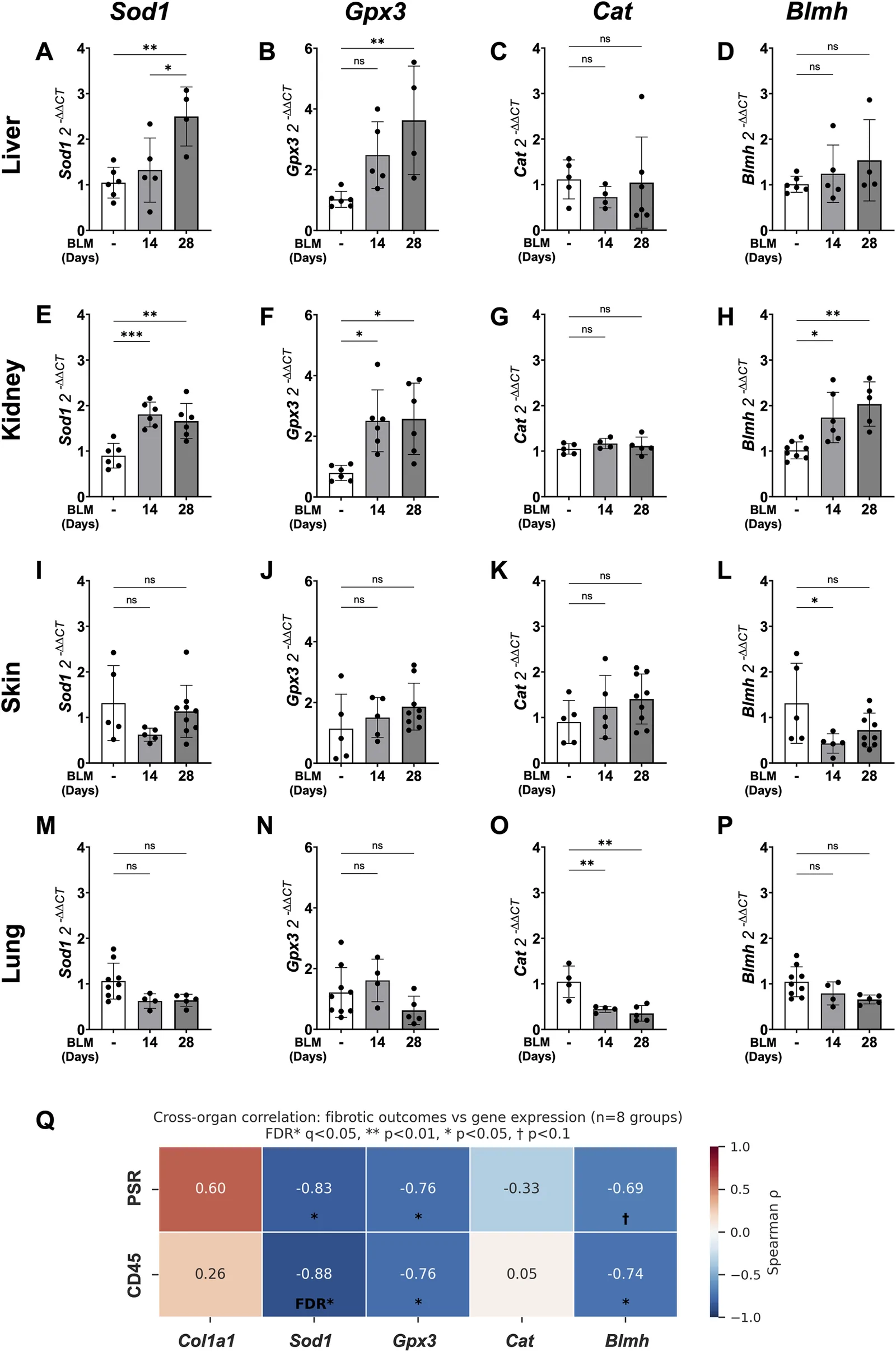
Tissue-specific antioxidant and bleomycin hydrolase expression signatures following systemic BLM exposure. Relative mRNA expression of the antioxidant enzymes *Sod1*, *Gpx3*, and *Cat*, together with *Blmh*, in the **(A–D)** liver, **(E–H)** kidneys, **(I–L)** skin, and **(M–P)** lungs of PBS-MP control and BLM-MP mice at D14 and D28 following BLM administration. Gene expression was quantified by RT-qPCR using the ΔΔCt method and is expressed as fold change relative to PBS-MP controls. Data are presented as mean ± SD. (Q) Cross-organ Spearman correlation matrix illustrating the relationship between collagen deposition (PSR-positive area), CD45^+^ cell infiltration, and tissue-specific gene-expression fold changes across organ/timepoint groups (n = 8 organ × timepoint combinations). Correlation coefficients (ρ) are color-coded according to strength and direction of association. Statistical significance is indicated as follows: ns, not significant; *p < 0.05, **p < 0.01, ***p < 0.001. FDR* indicates correlations remaining significant after Benjamini–Hochberg multiple-testing correction (q < 0.05).

To investigate whether antioxidant responses differed among organs following systemic BLM exposure, expression of the antioxidant enzymes superoxide dismutase (SOD1), glutathione peroxidase (GPX3), and catalase (CAT) was quantified in the liver, kidneys, skin and lungs. These genes were selected based on previously reported tissue-specific antioxidant expression profiles in healthy mice (Kim et al., 2018). In the liver, *Sod1* and *Gpx3* transcript levels increased at D28 but not D14 in BLM-MP mice (*Sod1*, PBS vs. BLM D14 vs. BLM D28, n=4−6 mean ± SD: 1.05 ± 0.34 vs. 1.32 ± 0.70 vs. 2.50 ±0.65 . *Gpx3*, PBS vs. BLM D14 vs. BLM D28, n=4−6 mean ± SD: 1.03 ± 0.26 vs. 2.48 ± 1.10 vs. 3.63 ±1.79 ) (Figures 3A,B). Similarly, both genes were upregulated in the kidneys at D14 and remained elevated at D28 (*Sod1*, PBS vs. BLM D14 vs. BLM D28, n=6 mean ± SD: 0.90 ± 0.27 vs. 1.81 ± 0.27 vs. 1.66 ±0.39 . *Gpx3*, PBS vs. BLM D14 vs. BLM D28, n=6 mean ± SD: 0.79 ± 0.25 vs. 2.51 ± 1.02 vs. 2.58 ±1.18 ) (Figures 3E,F).

In contrast, no significant changes in *Sod1* or *Gpx3* expression were observed in the skin of BLM-MP mice (*Sod1*, PBS vs. BLM D14 vs. BLM D28, n=5−7 mean ± SD: 1.32 ± 0.82 vs. 0.62 ± 0.14 vs. 1.26 ±0.57 . *Gpx3*, PBS vs. BLM D14 vs. BLM D28, n=5−7 mean ± SD: 1.14 ± 1.13 vs. 1.50 ± 0.66 vs. 1.90 ±0.88 ) (Figures 3I,J). In the lungs, *Sod1* expression was reduced at both at D14 and D28, while *Gpx3* decreased at D28 (*Sod1*, PBS vs. BLM D14 vs. BLM D28, n=4−9 mean ± SD: 1.06 ± 0.39 vs. 0.62 ± 0.16 vs. 0.64 ±0.13 . *Gpx3*, PBS vs. BLM D14 vs. BLM D28, n=4−9 mean ± SD: 1.21 ± 0.82 vs. 1.61 ± 0.70 vs. 0.62 ±0.47 ) (Figures 3M,N). *Cat* expression after BLM exposure remained stable across organs (Figures 3C,G,K) except in the lungs, where transcript levels decreased approximately two-fold at D14 and remained suppressed at D28 (*Cat*, PBS vs. BLM D14 vs. BLM D28, n=4−5 mean ± SD: 1.05 ± 0.34 vs. 0.44 ± 0.07 vs. 0.34 ±0.17 ) (Figure 3O). Together, these findings indicate that inflammatory responses vary substantially among organs following systemic BLM exposure and may contribute to differences in fibrotic remodeling. Fibrosis-resistant tissues exhibited preserved or increased antioxidant gene expression, whereas fibrosis-prone tissues demonstrated reduced or absent induction of these pathways.

### 3.4 Tissue-specific *Blmh* expression differs across organs following BLM exposure

To investigate whether differential BLM inactivation contributes to tissue susceptibility to fibrosis, expression of the BLM-inactivating enzyme (BLMH) was assessed across organs. *Blmh* transcript levels were unchanged in the liver of BLM-MP mice (PBS vs. BLM D14 vs. BLM D28, n = 4–6 mean ± SD: 1.01 ± 0.18 vs. 1.24 ± 0.63 vs. 1.54 ± 0.89) (Figure 3D), but significantly increased in the kidneys at both D14 and D28 (PBS vs. BLM D14 vs. BLM D28, n = 5–8 mean ± SD: 1.02 ± 0.19 vs. 1.74 ± 0.55 vs. 2.03 ± 0.49) (Figure 3H). In contrast, skin *Blmh* expression decreased approximately three-fold at D14, with a downward trend at D28 (PBS vs. BLM D14 vs. BLM D28, n = 5–7 mean ± SD: 1.31 ± 0.88 vs. 0.43 ± 0.21 vs. 0.61 ± 0.31), suggesting impaired BLM detoxification capacity (Figure 3L). A similar trend toward reduced *Blmh* expression was observed in the lungs at D28, although differences did not reach statistical significance (PBS vs. BLM D14 vs. BLM D28, n = 4–9 mean ± SD: 1.04 ± 0.32 vs. 0.79 ± 0.25 vs. 0.66 ± 0.10) (Figure 3P). Together, these findings suggest that *Blmh* expression exhibits distinct tissue-specific patterns following systemic BLM exposure and is reduced in organs that develop more extensive fibrotic remodeling.

### 3.5 Tissue-specific antioxidant and *Blmh* signatures are associated with inflammatory and fibrotic remodeling

To determine whether tissue-specific molecular responses were associated with inflammatory and fibrotic outcomes, we correlated antioxidant genes and *Blmh* expression with collagen deposition and CD45^+^ cell infiltration across organs and timepoints (Figure 3Q). Lower Sod1 expression was associated with both greater inflammatory infiltration (rho = −0.88, p = 0.004, FDR-adjusted q = 0.039) and increased collagen deposition (rho = −0.83, p = 0.010). Similar inverse associations were observed for *Gpx3* (rho = −0.76, p = 0.028), whereas *Blmh* expression showed inverse relationships with inflammation (rho = −0.74, p = 0.037) and a trend toward an inverse association with collagen deposition (rho = −0.69, p = 0.058). and expression. In contrast, *Cat* expression was not associated with either inflammatory infiltration or collagen deposition (rho = −0.33) (Figure 3Q; Supplementary Table S2; Supplementary Table S3). Together, these findings identify a tissue-specific molecular signature characterized by reduced antioxidant and *Blmh* expression in organs exhibiting greater inflammatory and fibrotic remodeling following systemic BLM exposure.

## 4 Discussion

In this study, we demonstrate that systemic BLM exposure elicits strikingly distinct inflammatory, fibrotic, and molecular responses across organs. While the skin and lungs developed progressive fibrosis, the kidneys exhibited only mild focal interstitial remodeling and the liver remained largely resistant. Importantly, these divergent tissue responses were accompanied by specific patterns of antioxidant enzyme and *Blmh* expression, identifying a molecular signature associated with inflammatory and fibrotic remodeling rather than a uniform response to systemic injury. Although the present study does not establish causality, these findings suggest that intrinsic tissue-specific stress response programs may contribute to the heterogeneous pattern of organ involvement observed in SSc and provide a framework for future mechanistic investigations.

The BLM-MP model reproduces several hallmark features of SSc, including subpleural interstitial lung fibrosis and progressive dermal fibrosis with lipoatrophy (Varga and Trojanowska, 2008; Lee et al., 2014). Our work showed that systemic BLM does not induce equivalent pathological responses across organs. This parallels the clinical heterogeneity of SSc, in which lungs and skin are preferentially affected. Although the model does not reproduce characteristic vascular lesions of SSc (Cole et al., 2023), it provides a valuable experimental platform to investigate mechanisms underlying tissue-selective fibrotic remodeling.

Early inflammatory responses are considered important initiators of fibrogenesis in both experimental fibrosis and SSc (Baffert et al., 2026; Distler et al., 2026; Jarnagin et al., 2026). Consistent with this concept, increased dermal CD45^+^ cell infiltration preceded maximal collagen accumulation in the skin, supporting the notion that inflammatory recruitment occurs during the early stages of tissue remodeling. In contrast, the liver and kidneys, showed little inflammatory infiltration paralleling their limited fibrotic responses. Pulmonary CD45^+^ cell frequencies were numerically higher but not statistically significant following BLM exposure. Together, these findings suggest that inflammatory response differs substantially among organs following systemic BLM exposure and may contribute to divergent fibrotic trajectories.

Oxidative stress has long been recognized as a central mediator of both BLM toxicity and SSc pathogenesis (Svegliati et al., 2005; Hecker et al., 2009; Jain et al., 2013; Allawzi et al., 2019; Kikuchi et al., 2010; Richter and Kietzmann, 2016; Doridot et al., 2019). However, less is known about the mechanisms underlying the tissue-specific responses to oxidative stress following a common systemic insult. Our findings suggest that the magnitude and direction of antioxidant responses vary markedly across organs, with fibrosis-resistant tissues mounting adaptive increases in *Sod1* and *Gpx3* expression, whereas fibrosis-prone tissues exhibited reduced or absent antioxidant induction. Although transcript expression does not directly reflect enzyme activity or oxidative burden, these observations support the concept that tissue-specific redox programs may influence how individual organs respond to chronic injury. Future studies integrating direct measurements of reactive oxygen species, antioxidant enzyme activity, and cell-specific transcriptomics will be important to determine whether these molecular signatures actively regulate tissue remodeling or simply reflect disease progression.

Alongside antioxidant responses, BLMH emerged as a second component of the molecular profile associated with tissue-specific fibrosis. Beyond antioxidant defenses, the capacity to metabolize BLM itself is likely to influence tissue exposure to oxidative injury. BLMH, the principal enzyme responsible for BLM inactivation, is widely expressed in mammalian tissues but displays relatively low pulmonary expression compared with the skin (Kamata et al., 2009; Jóna et al., 2016; Crnovcic et al., 2018). Experimental BLMH deficiency has been associated with worsened pulmonary injury and dermatitis following BLM exposure (Schwartz et al., 1999; Riise et al., 2019). Moreover, high doses of BLM may directly suppress BLMH activity (Filderman et al., 1988), raising the possibility of a feed-forward mechanism that further enhances tissue susceptibility to oxidative injury. In the present study, whereas renal *Blmh* expression increased following BLM administration, skin and lung tissues demonstrated reduced expression, potentially prolonging local exposure to the drug and its downstream oxidative effects. Previous studies have demonstrated that BLMH deficiency increases susceptibility to BLM-induced tissue injury, while alterations in BLMH have also been implicated in inflammatory regulation and wound repair (Riise et al., 2019). Increased renal *Blmh* expression may additionally reflect the role of the kidneys in BLM excretion and metabolism (Dorr, 1992). Nevertheless, the present study cannot determine whether reduced *Blmh* expression contributes directly to fibrosis or instead reflects tissue-specific responses to injury. Rather, our findings position BLMH as part of a broader molecular program associated with divergent tissue remodeling that warrants further mechanistic investigation.

Several limitations should be acknowledged. First, this study relied primarily on bulk tissue transcript analysis and histopathological assessment, precluding identification of the specific cellular populations driving the observed inflammatory, antioxidant, and BLMH responses. Second, oxidative stress was inferred from antioxidant gene expression rather than direct quantification of ROS, oxidative damage or antioxidant enzyme activity. Similarly, BLMH activity was not quantified. Third, correlation analyses cannot establish causal relationships between tissue-specific molecular signatures and fibrotic outcomes. Finally, only male mice were studied. Given the marked female predominance of SSc, sex-specific differences in antioxidant responses, inflammatory pathways and fibrosis susceptibility should be investigated in future studies to determine the generalizability of these findings. Nevertheless, this study provides a systematic characterization of tissue-specific susceptibility to fibrosis in the BLM-MP model and identifies antioxidant responses and BLM detoxification pathways as potential contributors of organ vulnerability.

In conclusion, systemic BLM exposure induces distinct inflammatory, fibrotic, and molecular responses across organs. Fibrosis-prone tissues were characterized by reduced antioxidant and *Blmh* expression, whereas fibrosis-resistant organs exhibited preserved or adaptive stress-response programs. Together, these findings identify a molecular profile associated with divergent fibrotic responses and generate testable hypotheses regarding mechanisms underlying organ-selective fibrosis. These observations broaden the utility of the BLM-MP model and provide a foundation for future studies investigating oxidative stress and BLMH pathways as potential therapeutic targets in SSc.

## Data Availability

The original contributions presented in the study are included in the article/Supplementary Material, further inquiries can be directed to the corresponding author.
