# Supplementary material for: Tissue-specific oxidative stress and bleomycin hydrolase signatures are associated with organ-specific fibrotic responses in a mouse model of systemic sclerosis
Source: Front Cell Dev Biol. 2026 Aug 18;14:1888823. doi: 10.3389/fcell.2026.1888823 (PMC13529668; doi:10.3389/fcell.2026.1888823)
Supplement: Supplementary file 1 [file Table1.docx]

Supplementary table 1. qRT-PCR primer sequences

| Gene Name | Forward sequence | Reverse sequence |
| --- | --- | --- |
| *Col1a1* | 5’- ACCTTCCTGCGCCTAATGTC-3’ | 5’- AGTTCC GGTGTGACTCGTG-3’ |
| *Sod1* | 5′-CAG AAGGCAAGCGGTGAAC-3′ | 5′-CAGCCTTGTGTATTGTCCCCATA-3’ |
| *Gpx3* | 5′- ATCCTGCCTTCTGTCCCTGCTC -3’ | 5′- TGGTGAGGGCTCCATACTCGTA -3’ |
| *Cat* | 5’GGACGCTCAGCTTTTCATTC -3’ | 5’- TTGTCCAGAAGAGCCTGGAT-3’ |
| *Blmh* | 5’-TCGCATACGACAGAGGCTACCA-3’ | 5’- CTTTGGTGGCTCCACTGTGTAC-3’ |
| *Sdha* | 5’-GAGATACGCACCTGTTGCCAAG-3’ | 5’-GGTAGACGTGATCTTTCTCAGGG-3’ |
| *Hprt* | 5’-CTGGTGAAAAGGACCTCTCGAAG-3’ | 5’-CCAGTTTCACTAATGACACAAAC-3’ |

*Col1a1: Collagen 1a1, Sod1:* *Superoxide dismutase 1, Gpx3:* *Glutathione peroxidase 3, Cat: Catalase, Blmh: Bleomycin hydrolase, Sdha: Succinate dehydrogenase complex subunit A, Hprt: Hypoxanthine phospho-ribosyl-transferase.*

Supplementary table 2. Fold-change per organ - timepoint (BLM / PBS control).

| **Organ** | **Day** | **PSR** | **CD45** | **Col1a1** | **Sod1** | **Gpx3** | **Cat** | **Blmh** |
| --- | --- | --- | --- | --- | --- | --- | --- | --- |
| **Liver** | 14 | 1.55 | 0.71 | 0.39 | 1.26 | 2.42 | 0.65 | 1.23 |
| **Liver** | 28 | 1.03 | 0.69 | 0.65 | 2.39 | 2.00 | 0.94 | 1.52 |
| **Kidney** | 14 | 1.34 | 0.76 | 0.72 | 2.01 | 3.17 | 1.11 | 1.71 |
| **Kidney** | 28 | 1.50 | 0.73 | 0.79 | 1.85 | 2.20 | 1.06 | 2.00 |
| **Skin** | 14 | 1.83 | 2.30 | 0.18 | 0.47 | 1.32 | 1.37 | 0.33 |
| **Skin** | 28 | 2.46 | 1.67 | 2.10 | 0.86 | 1.64 | 1.56 | 0.55 |
| **Lung** | 14 | 2.58 | 1.73 | 6.07 | 0.59 | 1.33 | 0.42 | 0.76 |
| **Lung** | 28 | 6.46 | 2.13 | 2.48 | 0.60 | 0.87 | 0.34 | 0.63 |

*All values are fold-changes relative to the PBS control of the same organ.*

Supplementary table 3. Cross-organ Spearman correlations (n = 8 organ x timepoint groups).

| **Outcome** | **Predictor** | **Spearman rho** | **p-value** | **BH q-value** | **Sig** |
| --- | --- | --- | --- | --- | --- |
| **CD45** | Sod1 | -0.88 | 0.004 | 0.039 | FDR* |
| **CD45** | Gpx3 | -0.76 | 0.028 | 0.070 | * |
| **CD45** | Blmh | -0.74 | 0.037 | 0.073 | * |
| **CD45** | Col1a1 | +0.26 | 0.531 | 0.590 | ns |
| **CD45** | Cat | +0.05 | 0.911 | 0.911 | ns |
| **PSR** | Sod1 | -0.83 | 0.010 | 0.051 | * |
| **PSR** | Gpx3 | -0.76 | 0.028 | 0.070 | * |
| **PSR** | Blmh | -0.69 | 0.058 | 0.097 | ƚ |
| **PSR** | Col1a1 | +0.60 | 0.120 | 0.171 | ns |
| **PSR** | Cat | -0.33 | 0.420 | 0.525 | ns |

*All correlations are fibrotic outcome (PSR or CD45) fold-change vs the indicated gene's fold-change across n = 8 organ x timepoint groups. FDR* = survives Benjamini-Hochberg q<0.05; ** p<0.01; * p<0.05; ƚ p<0.1; ns = not significant.*
